# PP19 Time And Cost Savings Of Machine Learning And Artificial Intelligence (AI) In Systematic Reviews: A Case Study

**DOI:** 10.1017/S0266462324001922

**Published:** 2025-01-07

**Authors:** Anne Littlewood, Rachael McCool, Mary Edwards, Lavinia Ferrante di Ruffano

## Abstract

**Introduction:**

Conducting a systematic review (SR) of clinical trials is labor-intensive and expensive. However, existing open-source content can be used to develop custom machine learning tools suited to the workflow of individual organizations. This case study details the potential of a bespoke tool developed by York Health Economics Consortium (YHEC) for reducing the time and cost involved in producing an SR.

**Methods:**

RESbot is a flexible, stand-alone machine learning tool created using an extensively tested open-source dataset developed by Cochrane. The tool identifies randomized controlled trials (RCTs) from a large corpus of records. It has a user interface and inputs/outputs to fit into the company’s existing workflow at any stage. RESbot has two settings. The “sensitive” setting identifies a higher number of possible RCTs with a lower risk of missing eligible studies, while the “precise” setting is more focused. For both settings, we estimated the reduction in resources required for record screening in two examples of RCT-only reviews.

**Results:**

Scoping searches in MEDLINE were conducted for SRs of RCTs in femoropopliteal artery diseases (FAD) and postpartum depression (PD). The results were run through RESbot. For the FAD SR, 1,444 references were retrieved, with the sensitive and precise RESbot settings reducing the record set by 38 percent and 64 percent, respectively. For the PD SR, a record set of 2,153 records was reduced by 25 percent and 41 percent, respectively. Resource savings offered by RESbot vary depending on subject but may reduce the time taken to screen records by up to 64 percent, with a subsequent reduction in cost to the organization commissioning the SR.

**Conclusions:**

The use of bespoke machine learning tools in SR production has the potential to reduce the time and staff costs involved in producing a review. This case study tested the effect on a small number of records, but for larger reviews retrieving tens of thousands of records, reductions in time and costs can be very significant.

